# Sequence Alignment-Based Prediction of Myosin 7A: Structural Implications and Protein Interactions

**DOI:** 10.3390/ijms25063365

**Published:** 2024-03-16

**Authors:** Chan Jong Yu, Yoon Ho Park, Bumhan Ryu, Hyun Suk Jung

**Affiliations:** 1 Division of Chemistry & Biochemistry, Department of Biochemistry, College of Natural Sciences, Kangwon National University, Chuncheon 24341, Republic of Korea; cjyu@kangwon.ac.kr (C.J.Y.); yhpark99@kangwon.ac.kr (Y.H.P.); 2 Research Solution Center, Institute for Basic Science, Daejeon 34126, Republic of Korea

**Keywords:** multiple sequence alignment, sequence-based structure prediction, actomyosin complex, pliant region

## Abstract

Myosin, a superfamily of motor proteins, obtain the energy they require for movement from ATP hydrolysis to perform various functions by binding to actin filaments. Extensive studies have clarified the diverse functions performed by the different isoforms of myosin. However, the unavailability of resolved structures has made it difficult to understand the way in which their mechanochemical cycle and structural diversity give rise to distinct functional properties. With this study, we seek to further our understanding of the structural organization of the myosin 7A motor domain by modeling the tertiary structure of myosin 7A based on its primary sequence. Multiple sequence alignment and a comparison of the models of different myosin isoforms and myosin 7A not only enabled us to identify highly conserved nucleotide binding sites but also to predict actin binding sites. In addition, the actomyosin-7A complex was predicted from the protein–protein interaction model, from which the core interface sites of actin and the myosin 7A motor domain were defined. Finally, sequence alignment and the comparison of models were used to suggest the possibility of a pliant region existing between the converter domain and lever arm of myosin 7A. The results of this study provide insights into the structure of myosin 7A that could serve as a framework for higher resolution studies in future.

## 1. Introduction

Myosin, a superfamily of motor proteins composed of numerous isoforms, moves by binding to the actin filament using energy generated via an ATP-dependent mechanism [1,2]. Structurally, myosin is composed of a motor, neck, and tail domain, with the motor domain comprising the actin and ATP binding sites [3,4]. Upon binding to ATP, this motor domain undergoes ATP hydrolysis to create a cross-bridge between the myosin motor domain and actin filament in the ADP-Pi state. The release of Pi from this state causes a force that generates a powerstroke step. The converter domain translates small conformational changes (at the angstrom scale) in the motor domain into large movements of the lever arm, which execute the powerstroke [5,6,7]. The lever arm can undertake larger movements during the powerstroke process depending on the number of IQ motifs and the presence or absence of pliant regions [8,9]. A pliant region was first proposed to exist in the scallop myosin by comparing the structural differences between the nucleotide-free state and MgADP-Vi (vanadate) states. The pliant region was defined as the area between the converter domain and lever arm, where a significant change in the angle was observed in the region of the alpha helix [8]. Based on this research, the pliant region of myosin has been studied by many and is known to affect the step size and velocity of motility [10,11,12,13,14].

Myosin isoforms play an important role in many cell functions, such as cell movement, muscle contraction, and intracellular transport through movement, and dysregulation or mutations in myosin cause significant problems for cell function and human health [15,16,17]. Among the different classes of myosin, myosin 7A is an unconventional motor protein involved in the growth and maintenance of stereocilia. Mutations in the myosin 7A gene lead to the genetic disorder known as Usher syndrome type 1B. Infants with this condition are born with profound hearing loss or deafness and may experience balance issues, leading to delayed walking until around 18 months of age. Additionally, vision loss typically begins around the age of 10 and worsens over time, starting with night blindness and progressing to severe visual impairment over several years. Currently, there is no cure for Usher syndrome, and management using hearing aids, cochlear implants, visual aids, and similar devices is the only available approach. A functional study utilizing shaker-1 mice with mutated myosin 7A genes has revealed the crucial role of myosin 7A in the arrangement of hair bundles within stereocilia and the survival of retinal cells [18,19,20,21,22]. Myosin isoforms linked to human disorders are being actively studied from a functional perspective, and structural investigations are underway to completely understand their activities. Therefore, current advances in structural determination technologies have paved the way to resolving the high-resolution structures of myosin as well as those of actomyosin complexes; however, many isoforms of myosin are still unknown. This could be attributed to the difficulty in purifying highly homogeneous proteins needed for structural determination and the time-consuming nature of the entire process.

Homology modeling, also known as sequence-based structure prediction, is a computer-based technique used to construct 3D models of proteins or other macromolecules by utilizing the known 3D structure of a related protein with a similar amino acid sequence. This technique is based on the principle that the structure and function of a protein are mostly determined by its amino acid sequence, and that proteins with similar sequences are likely to have similar structures [23,24,25]. The amino acid sequence is determined using the Edman degradation (a direct sequencing method) and mass spectrometry (an indirect sequencing method), and sequence alignment can be used to identify the similarities between two or more protein sequences [26,27]. The information obtained from the aligned sequences can be used to investigate the evolutionary relationships and hence predict the structure and function [28,29]. This technique is based on an algorithm that takes into account various factors, such as the physicochemical properties and interactions of amino acids, and has the advantage of saving time and cost in determining protein structures [23,24,25]. However, because accurate predictions are not always possible, this method can facilitate our understanding of proteins until the actual structural determination is completed.

Most proteins do not function alone and function or regulate by interacting with other proteins. This has led to the widespread adoption of computational approaches for predicting protein–protein interaction (PPI). PPI refers to highly specific physical contact between protein molecules, including electrostatic forces, hydrogen bonding, and the hydrophobic effect, and plays important roles in various biological processes such as biochemical reactions, signaling, cell cycle control, and neurotransmission [30,31,32,33,34]. Techniques for predicting PPIs include docking and molecular dynamics simulations, which help to understand biological systems and disease mechanisms by structurally predicting the interfaces at which interaction takes place [35,36].

In this study, we investigated the key active sites of myosin 7A through sequence-based computational analysis. Specifically, we focused on providing structural insights into myosin 7A using sequence alignment, homology modeling, and molecular docking. Through these methods, we propose the nucleotide binding sites, actin binding sites, and the core interface between actin and myosin 7A as the key active sites of myosin 7A. Additionally, we suggest the potential existence of a pliant region. The approaches employed in this study not only provide essential structural insights into myosin isoforms with unknown structures but also can serve as a foundation for high-resolution structural studies.

## 2. Results

### 2.1. Conservation of Amino Acid Sequence in the Nucleotide-Binding Sites of Myosin 7A Motors

**Figure 1:**
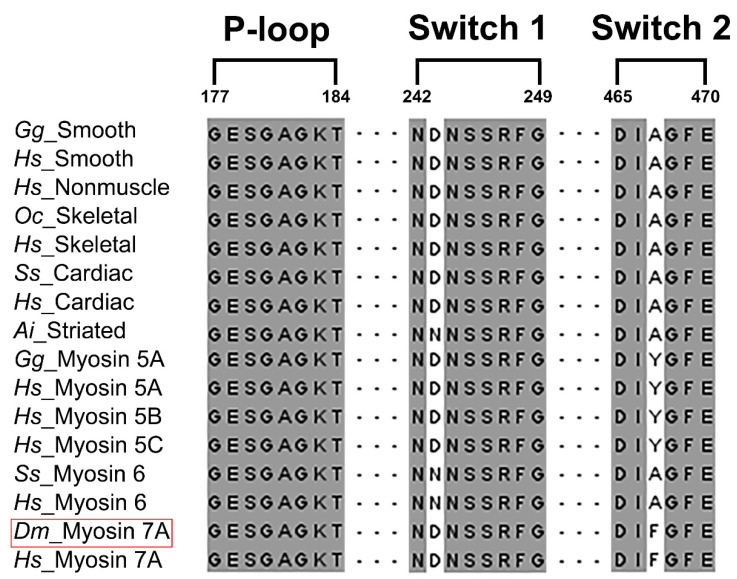
Amino acid sequence alignment of nucleotide-binding sites from myosin 7A (in red box) and other myosin isoforms. Three loops (P-loop, switch-1, switch-2) were selected from the full sequence alignment of myosin 7A and other myosin isoforms (Supplementary Figure S1) [41,42]. Conserved residues are highlighted in gray. Species codes: *Gg Gallus gallus*; *Hs Homo sapiens*; *Oc Oryctolagus cuniculus*; *Ss Sus scrofa*; *Ai Argopecten irradians*; *Dm Drosophila melanogaster*.

**Figure 2:**
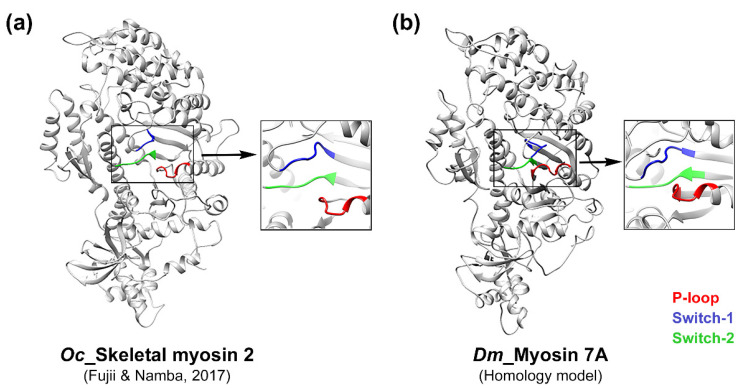
Structural comparison of the nucleotide-binding sites of skeletal myosin 2 and myosin 7A homology model. (**a**) Model of skeletal myosin 2 based on the skeletal actomyosin complex (PDB: 5H53) [43], adapted after excluding actin from the structure. (**b**) Homology model of myosin 7A (Supplementary Figure S2) [44,45]. The nucleotide binding sites from the model are indicated in specific colors. Species codes: *Oc Oryctolagus cuniculus*; *Dm Drosophila melanogaster*.

Myosin performs numerous functions by moving using energy generated through ATP hydrolysis [1,2]. Previous studies have shown that nucleotide binding sites exist in the motor domain of myosin, showing a high level of sequence conservation [37,38,39,40]. However, the sequence identity of myosin 7A with other myosin isoforms does not exceed 42%. Therefore, multiple sequence alignments and model comparisons were performed to predict the nucleotide-binding sites of myosin 7A (Figure 1, Figure 2, Figures S1 and S2). Three highly conserved regions were discovered using multiple sequence alignment, and the regions were identified as nucleotide binding sites in other myosin isoforms (Figure 1). A comparison of skeletal myosin 2 and homology model of myosin 7A also revealed the location and structure of binding sites to be highly similar (Figure 2a,b). This could indicate that P-loop (G156–T163), switch-1 (N205–G212), and switch-2 (D431–E436) form a part of the nucleotide-binding sites in myosin 7A.

### 2.2. Prediction of the Actin Binding Sites in Actomyosin-7A Complex

**Figure 3:**
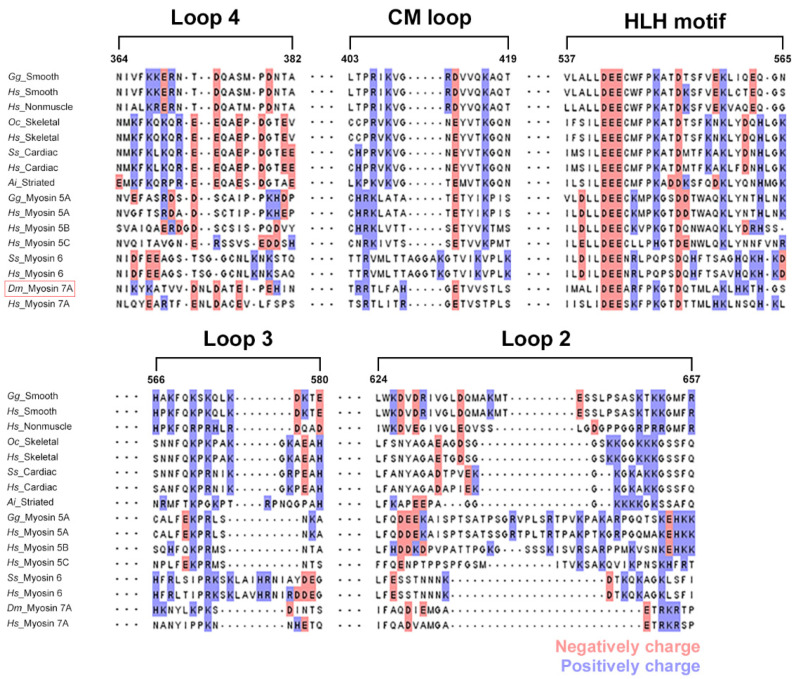
Comparison of the amino acid sequence of the actin binding interface between myosin 7A (in red box) and other myosin isoforms. The myosin 7A actin binding sites were predicted using sequence alignment. Five regions (loop-4, CM loop, HLH motif, loop-3, and loop-2) were selected from the full sequence alignment of myosin 7A and other myosin isoforms (Supplementary Figure S1). The number of residues corresponds to the original residues from skeletal myosin 2. Positively and negatively charged residues are displayed in red and blue, respectively. Species codes: *Gg Gallus gallus*; *Hs Homo sapiens*; *Oc Oryctolagus cuniculus*; *Ss Sus scrofa*; *Ai Argopecten irradians*; *Dm Drosophila melanogaster*.

**Figure 4:**
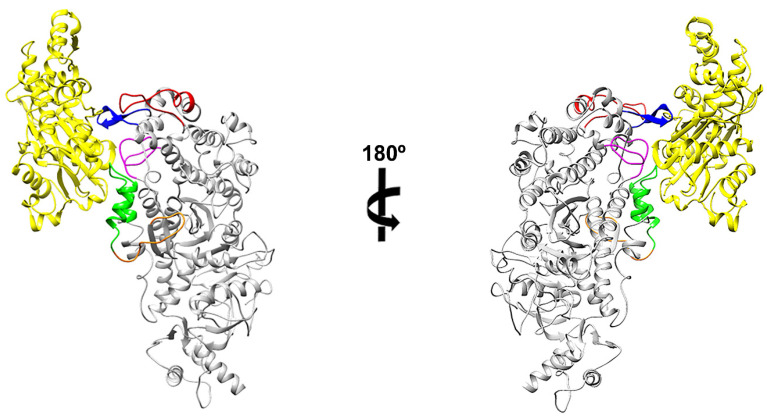
Protein–protein interaction prediction model between actin and myosin 7A. Model developed in this study: actomyosin-7A created from the myosin 7A homology model with alpha-skeletal actin (HDOCK server) [55]. Five actin binding sites predicted by sequence alignment are represented as different colors. Actin: yellow, loop-4: Red, CM loop: blue, HLH motif: green, loop-3: orange, loop-2: magenta.

With recent advances in cryo-EM technology, structural research on the interaction between actin and myosin is being actively conducted to understand the mechanism of myosin movement that uses filamentous actin as a track. When analyzing actomyosin models with known structures, characteristic structural differences exist depending on myosin isoforms. However, most actomyosin complexes have five actin binding sites, and each site has a similar secondary structure [43,46,47,48,49,50,51]. Because the structural similarity of actin binding sites has been observed, the sites where myosin 7A binds to actin could be predicted through sequence alignment. Electrostatic steering enables the initial binding of actin and myosin during the ATPase cycle, prompting the highlighting of charged residues within the actin binding sites using coloration [52,53,54]. The actin binding sites of myosin 7A were predicted to be loop-4 (N328–N349), cardiomyopathy loop (T369–S385), helix–loop–helix motif (I500–S528), loop-3 (H529–S542), and loop-2 (I586–P602) (Figure 3 and Figure S1). The structure of the actomyosin-7A complex was recreated using a protein–protein interaction prediction approach to confirm that the actin binding sites in the five regions predicted by sequence alignment are structurally located at a distance that can interact with actin. These predicted five actin binding sites from the structure of the created complex may be positioned structurally close enough to allow the possibility of interaction with actin (Figure 4).

### 2.3. Analysis of Myosin Motors to Define the Core Sites of the Actomyosin-7A Interface

**Figure 5:**
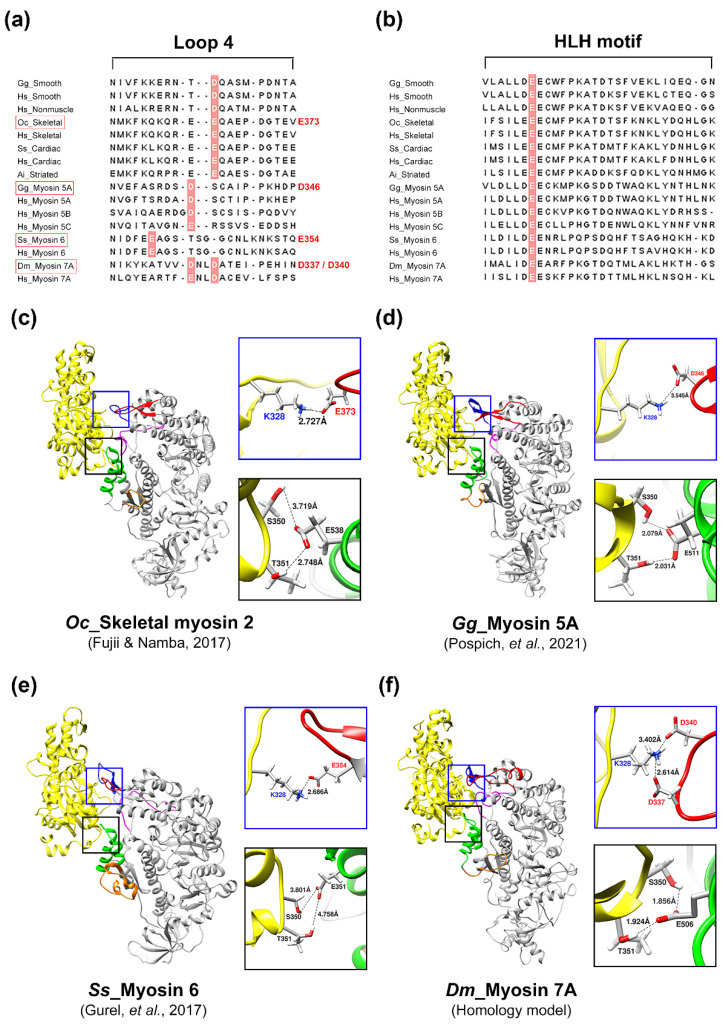
Predicted interface of the actomyosin-7A complex. Sequence alignment (**a**) of the loop-4 region of different myosin isoforms and (**b**) of the HLH motif. Note that the sequences of the (**c**–**f**) models have been indicated as red boxes from (**a**). Core residues involved in actin binding for different myosin isoforms are shown in red. The blue boxes show an enlargement of the electrostatic interactions in loop-4 of the different myosin isoforms with actin residue K328 (**c**–**f**). The black box presents an enlargement of the hydrogen bonding between the HLH motif of the myosin isoforms with actin residues S350 and T351 (**c**–**f**). The models (**c**–**e**) were extracted from the deposited actomyosin complex structures, i.e., actomyosin-2 skeletal (PDB: 5H53) [43], actomyosin-5A (PDB: 7PLT) [48], and actomyosin-6 (PDB: 6BNP) [51]. (**f**) Model developed in this study: actomyosin-7A created from the myosin 7A homology model with alpha-skeletal actin protein–protein interaction prediction (HDOCK server). Note that similar interactions were also observed when using the AlphaFold2_multimer “https://github.com/sokrypton/ColabFold (accessed on 11 March 2024)” (Supplementary Figure S3). Actin: yellow, loop-4: Red, CM loop: blue, HLH motif: green, loop-3: orange, loop-2: magenta. Species codes: *Gg Gallus gallus*; *Hs Homo sapiens*; *Oc Oryctolagus cuniculus*; *Ss Sus scrofa*; *Ai Argopecten irradians*; *Dm Drosophila melanogaster*.

The actin binding sites of myosin isoforms have similar secondary structures and exist in similar positions, but structural differences resulting from sequence differences between isoforms result in distinctive interactions with actin [43,48,49,50,51]. Interpretation of the distinctive interaction between myosin 7A and actin through the analysis of predicted actomyosin-7A can be challenging. However, through comparison with actomyosin models whose structures have been identified, it was confirmed that there is a specific sequence of actin that exhibits the same interaction as most myosin isoforms (Figure 5c–f). K328 of actin interacts with a negatively charged residue of myosin loop-4 via electrostatic interactions whereas S350 and T351 of actin form hydrogen bonds with residues of the myosin helix-loop-helix motif (Figure 5a–e). Four of the residues of the myosin 7A loop-4 regions are negatively charged, and the predicted actomyosin-7A model suggests that D337 and D340 may electrostatically interact with actin K328 (Figure 5a,f). In addition, since the residue that forms hydrogen bonds with actins S350 and T351 is highly conserved in myosin isoforms, myosin 7A residue E506 is expected to engage in hydrogen bonding with these two actins (Figure 5b,f).

### 2.4. Suggestive Evidences for the Presence of a Pliant Region of Myosin 7A

**Figure 6:**
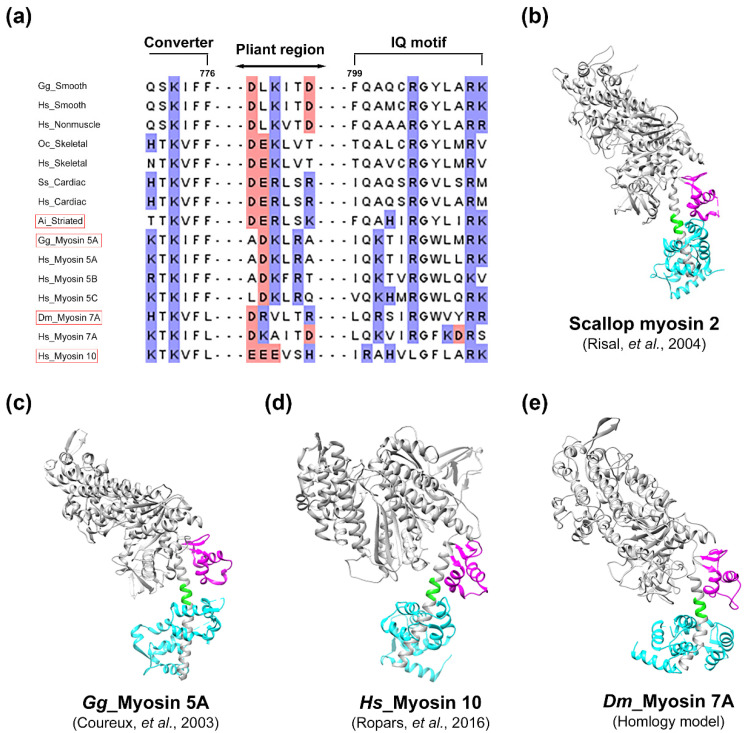
Prediction of the presence of a pliant region in myosin 7A. The pliant region was predicted to exist in myosin 7A using sequence alignment and model comparison. (**a**) Comparison between different aligned sequences revealed the existence of a pliant region at the junction between the converter domain and lever arm. Positively and negatively charged residues are shown in red and blue, respectively. (**b**–**d**) Three models reported to have pliant regions: scallop (*Argopecten irradians*) striated muscle myosin 2 nucleotide-free (PDB: 1SR6) [56], myosin 5A nucleotide-free (PDB: 1OE9) [57], and myosin 10 pre-powerstroke state (PDB: 5I0I) [58]. (**e**) Myosin 7A homology model developed in this study (Supplementary Figure S2). The converter domain, pliant region, and light chain are highlighted in magenta, green, and cyan, respectively. Note that the sequences of the (**b**–**e**) models have been indicated as red boxes from (**a**). Species codes: *Gg Gallus gallus*; *Hs Homo sapiens*; *Oc Oryctolagus cuniculus*; *Ss Sus scrofa*; *Ai Argopecten irradians*; *Dm Drosophila melanogaster*.

Considering that myosin 10 (42%) and 5A (39%) share the highest degree of identity with myosin 7A, the likely presence of a pliant region in myosin 7A was examined in depth (Figure 6). As a result of the multiple sequence alignment of the myosin isoforms, although the residue of the pliant region was found not to be highly conserved, the ratio of the charged residue was likely to be higher (Figure 6a). A comparison of the homology model of myosin 7A with the models of myosin isoforms that have pliant regions led to the observation of a structure composed of an alpha helix between the converter domain and the lever arm, indicating a high degree of structural similarity (Figure 6b–e). This seemed to indicate a higher possibility of the existence of a pliant region between the converter domain and the lever arm of myosin 7A.

## 3. Discussion

Myosin 7A is identified as the gene responsible for Usher syndrome type 1B [19,59]. It is expressed in actin-rich membrane protrusions found in the inner ear and retina, specifically in the hair bundles and the retinal pigment epithelium. Particularly in hair cells, myosin 7A utilizes linker proteins as binding partners, enabling the stereocilia to interconnect and form the hair bundle. Additionally, in the retinal pigment epithelium, myosin 7A interacts with opsins, phototransduction proteins, melanosomes, phagosomes, and other molecules, facilitating the distribution and transport of these proteins [60,61]. These findings imply that myosin 7A may play a role in transporting partner proteins to their proper locations through movement after binding.

To date, research on the functional aspects of myosin 7A, which has been the focus of investigation, still leaves important unresolved questions regarding the regulation of its functions. As structural changes and movements occur concurrently for functional performance, understanding the relationship between structure and function is crucial [5,6,7]. Active structural research is underway on various myosin isoforms; however, due to the discovered structural differences among myosin isoforms, independent structural studies specifically focusing on myosin 7A are necessary.

Thus, we employed different computational methodologies such as sequence alignment, homology modeling, and molecular docking to provide structural information about the active sites involved in myosin 7A at atomic level. These include the nucleotide binding sites, actin binding sites, and the core interface between actin and myosin 7A. Furthermore, we postulated the potential presence of a pliant region based on the sequence similarity, giving hints at a shared structural resemblance within the motor domain across various myosin isoforms. By the proposing the key active sites of myosin 7A, our study delivers a background on the structural composition of myosin 7A.

When myosin binds to actin or ATP, the salt bridge formed between switch-2 glutamic acid and switch-1 arginine in the nucleotide binding sites appears crucial for positioning the essential catalytic residues [62]. Through mutation studies involving the substitution of arginine, which forms the salt bridge, with alanine, a significant decrease in ATPase activity was detected [63]. While most myosin proteins possess highly conserved nucleotide binding sites, myosin 18A, with the substitution of switch-2 glutamic acid to asparagine, is implied to have low ATPase activity due to the inability to form the salt bridge [64]. On the other hand, myosin 7A is suggested to exhibit general ATPase activity since it possesses highly conserved nucleotide binding sites with no significant change in the sequence (Figure 1).

The initiation of the ATPase cycle leads to the initial weak binding between the actin N-terminus and the charged residues in myosin loop-2 during the pre-powerstroke state [50]. Subsequently, upon the release of Pi, the formation of the powerstroke and strong binding occurs between the actin filament and the HLH motif through hydrophobic interactions and interactions involving polar residues [65,66]. The highly conserved proline within the HLH motif, along with surrounding phenylalanine [49,50], leucine [51], and methionine [48], plays a central role in the hydrophobic interaction with actin. When these residues are mutated to alanine, actin-based motility is completely abolished or significantly reduced [54]. The binding between the HLH motif and the actin filament is stabilized by interactions among polar residues, and the glutamic acid centrally located among three highly conserved negatively charged residues in the HLH motif is noted to form hydrogen bonds with actin S350 and T351 [43,48,49,50,51,67]. When the specific glutamic acid is mutated, it has been reported that both ATPase activity and actin binding affinity decrease more than tenfold [52]. Therefore, the highly conserved E506 in myosin 7A is also proposed to form a crucial structural and functional interface with actin. Shifting the perspective to analyze the structural interaction with myosin isoforms based on actin reveals the observation of electrostatic interaction between actin K328 and the negatively charged residue in myosin loop-4 [43,46,47,48,49,50,51,67]. Selecting negatively charged residues in the loop-4 region of myosin 7A and analyzing the predicted structure of actomyosin-7A indicates that D337 and D340 may engage in electrostatic interaction with actin K328 (Figure 5). However, due to the structural flexibility inherent in loops, it is necessary to elucidate through high-resolution structural studies whether both proposed residues, D337 and D340, interact simultaneously or if only one of them participates in the interaction. The myosin lever arm performs one of the most essential functions in the myosin chemomechanical cycle by converting the chemical energy of ATP hydrolysis into physical motion, enabling the function of the myosin motor [6,7]. While the characteristics of the myosin motor domain are strongly conserved, the position of the lever arm can vary significantly between different isoforms of the same state [8,9]. This variability is attributed to the unique flexibility of the pliant region allowing the lever arm to assume different structures. Therefore, different angles of the lever arm during the ATPase cycle may arise from either active movement of the converter domain or passive bending at the pliant region. The pliant region enables myosin to have a variable step size, allowing it to choose multiple actin sites for binding [13]. Recently, mutational studies on the pliant region involving glutamine (D778), leucine (L781), and serine (S782) revealed their association with motor activity, the formation of the autoinhibited state, actin gliding velocity, and duty ratio [68]. The predicted pliant region of myosin 7A includes glutamine and leucine, and it contains threonine, which shares similar characteristics with serine (Figure 6). While the step size of *Drosophila* myosin 7A is approximately 30 nm [69], predicting a variable step size in the presence of a pliant region, it is also predicted to have a unique lever arm swing angle during the ATPase cycle. For a comprehensive understanding of myosin 7A, structural studies are necessary to confirm the presence of the pliant region.

Recent advancements in high-resolution structural elucidation techniques have made significant contributions to understanding the mechanisms underlying biological phenomena. However, the interpretation of structures requires considerable time and effort, and numerous challenging tasks still remain unresolved. In this study, we propose an approach utilizing sequence-based computational analysis to overcome these limitations. In conclusion, we have offered structural insights into the key active sites of the myosin 7A motor domain and suggested the potential presence of a flexible region. However, fully comprehending the distinctive structure of myosin 7A has limitations, especially in cases of highly flexible structures, imposing constraints on interpretation. Further high-resolution studies are needed to gain a more comprehensive understanding of the movement and regulatory mechanisms of myosin 7A.

## 4. Materials and Methods

### 4.1. Multiple Sequence Alignment and Analysis

Whole protein sequences of myosin isoforms were downloaded from the UniProt database “https://www.uniprot.org/ (accessed on 14 March 2023)”: myosin 2 smooth (*Gallus gallus*, UniProt-P10587), myosin 2 smooth (*Homo sapiens*, UniProt-P35749), myosin 2 nonmuscle (*Homo sapiens*, UniProt-Q7Z406), myosin 2 skeletal (*Oryctolagus cuniculus*, UniProt-Q9GJP9), myosin 2 skeletal (*Homo sapiens*, UniProt-Q9UKX3), myosin 2 cardiac (*Sus scrofa*, UniProt-P79293), myosin 2 cardiac (*Homo sapiens*, UniProt-P12883), myosin 2 scallop (*Argopecten irradians*, UniProt-P24733), myosin 5A (*Gallus gallus*, UniProt-Q02440), myosin 5A (*Homo sapiens*, UniProt-Q9Y4I1), myosin 5B (*Homo sapiens*, UniProt-Q9ULV0), myosin 5C (*Homo sapiens*, UniProt-Q9NQX4), myosin 6 (*Sus scrofa*, UniProt-Q29122), myosin 6 (*Homo sapiens*, UniProt-Q9UM54), myosin 7A (*Drosophila melanogaster*, UniProt-Q9V3Z6), myosin 7A (*Homo sapiens*, UniProt-Q13402), myosin 10 (*Homo sapiens*, UniProt-Q9HD67). Alignments were generated using T-Coffee “https://tcoffee.crg.eu/apps/tcoffee/do:expresso (accessed on 31 March 2023)” [41] and the ClustalW output format. The alignments were colored using Jalview “2.11.3.2, The Barton Group, University of Dundee, Scotland, UK” [42].

### 4.2. Model Building and Visualization

Homology modeling of myosin 7A was conducted using AlphaFold “https://alphafold.ebi.ac.uk/ (accessed on 13 December 2023)” [45], I-TASSER “https://zhanggroup.org/I-TASSER/ (accessed on 24 January 2024)” [23], and SWISS-MODEL “https://swissmodel.expasy.org/ (accessed on 15 June 2023)” [44]. Despite differences in software and reference choices, a high level of structural similarity was observed in the head domain of myosin 7A (Supplementary Figure S2). Among these, a myosin 7A model generated by the SWISS-MODEL software, which was created with reference to the extensively studied unconventional myosin 5A, was utilized. Using the HDOCK server [55] with default settings, we performed protein–protein docking simulation between the myosin 7A homology model and the alpha-skeletal actin from the skeletal actomyosin complex (PDB; 5H53), resulting in the generation of the actomyosin-7A complex. A suitable actomyosin-7A complex model was chosen from the numerous docking results, taking into account the highly conserved interaction sites between actin and myosin isoforms from the perspective of actin. All the proteins in the figures were visualized by UCSF chimera “1.16, Regents of the University of California, San Francisco, CA, USA” [70].

## Data Availability

The datasets used and/or analyzed during the current study are available from the corresponding author on reasonable request.

## Supplementary Materials

The following supporting information can be downloaded at: https://www.mdpi.com/article/10.3390/ijms25063365/s1.

## Abbreviations

ATP (adenosine triphosphate); ADP (adenosine diphosphate); Pi (inorganic phosphate); PPI (protein–protein interaction); 3D (three-dimensional).

## References

[1] Mermall V., Post P.L., Mooseker M.S. (1998). Unconventional myosins in cell movement, membrane traffic, and signal transduction. Science.

[2] Sellers J.R. (2000). Myosins: A diverse superfamily. Biochim. Biophys. Acta.

[3] Cope M.J., Whisstock J., Rayment I., Kendrick-Jones J. (1996). Conservation within the myosin motor domain: Implications for structure and function. Structure.

[4] Korn E.D. (2000). Coevolution of head, neck, and tail domains of myosin heavy chains. Proc. Natl. Acad. Sci. USA.

[5] Lymn R.W., Taylor E.W. (1970). Transient state phosphate production in the hydrolysis of nucleoside triphosphates by myosin. Biochemistry.

[6] Lymn R.W., Taylor E.W. (1971). Mechanism of adenosine triphosphate hydrolysis by actomyosin. Biochemistry.

[7] Okimoto N., Yamanaka K., Ueno J., Hata M., Hoshino T., Tsuda M. (2001). Theoretical studies of the ATP hydrolysis mechanism of myosin. Biophys. J..

[8] Houdusse A., Szent-Gyorgyi A.G., Cohen C. (2000). Three conformational states of scallop myosin S1. Proc. Natl. Acad. Sci. USA.

[9] Burgess S., Walker M., Wang F., Sellers J.R., White H.D., Knight P.J., Trinick J. (2002). The prepower stroke conformation of myosin V. J. Cell Biol..

[10] Gourinath S., Himmel D.M., Brown J.H., Reshetnikova L., Szent-Györgyi A.G., Cohen C. (2003). Crystal structure of scallop Myosin s1 in the pre-power stroke state to 2.6 a resolution: Flexibility and function in the head. Structure.

[11] Oke O.A., Burgess S.A., Forgacs E., Knight P.J., Sakamoto T., Sellers J.R., White H., Trinick J. (2010). Influence of lever structure on myosin 5a walking. Proc. Natl. Acad. Sci. USA.

[12] Ménétrey J., Bahloul A., Wells A.L., Yengo C.M., Morris C.A., Sweeney H.L., Houdusse A. (2005). The structure of the myosin VI motor reveals the mechanism of directionality reversal. Nature.

[13] Sun Y., Goldman Y.E. (2011). Lever-arm mechanics of processive myosins. Biophys. J..

[14] Ménétrey J., Isabet T., Ropars V., Mukherjea M., Pylypenko O., Liu X., Perez J., Vachette P., Sweeney H.L., Houdusse A.M. (2012). Processive steps in the reverse direction require uncoupling of the lead head lever arm of myosin VI. Mol. Cell.

[15] Coluccio L.M. (2020). Myosins and Disease. Adv. Exp. Med. Biol..

[16] Warrick H.M., Spudich J.A. (1987). Myosin structure and function in cell motility. Annu. Rev. Cell Biol..

[17] Brown M.E., Bridgman P.C. (2004). Myosin function in nervous and sensory systems. J. Neurobiol..

[18] Gibbs D., Kitamoto J., Williams D.S. (2003). Abnormal phagocytosis by retinal pigmented epithelium that lacks myosin VIIa, the Usher syndrome 1B protein. Proc. Natl. Acad. Sci. USA.

[19] Weil D., Blanchard S., Kaplan J., Guilford P., Gibson F., Walsh J., Mburu P., Varela A., Levilliers J., Weston M.D. (1995). Defective myosin VIIA gene responsible for Usher syndrome type 1B. Nature.

[20] Hasson T., Heintzelman M.B., Santos-Sacchi J., Corey D.P., Mooseker M.S. (1995). Expression in cochlea and retina of myosin VIIa, the gene product defective in Usher syndrome type 1B. Proc. Natl. Acad. Sci. USA.

[21] Hasson T., Gillespie P.G., Garcia J.A., MacDonald R.B., Zhao Y., Yee A.G., Mooseker M.S., Corey D.P. (1997). Unconventional myosins in inner-ear sensory epithelia. J. Cell Biol..

[22] Liu X., Ondek B., Williams D.S. (1998). Mutant myosin VIIa causes defective melanosome distribution in the RPE of shaker-1 mice. Nat. Genet..

[23] Zhang Y. (2008). I-TASSER server for protein 3D structure prediction. BMC Bioinform..

[24] Schwede T., Kopp J., Guex N., Peitsch M.C. (2003). SWISS-MODEL: An automated protein homology-modeling server. Nucleic Acids Res..

[25] Kim D.E., Chivian D., Baker D. (2004). Protein structure prediction and analysis using the Robetta server. Nucleic Acids Res..

[26] Edman P. (1949). A method for the determination of amino acid sequence in peptides. Arch. Biochem..

[27] Hunt D.F., Yates J.R., Shabanowitz J., Winston S., Hauer C.R. (1986). Protein sequencing by tandem mass spectrometry. Proc. Natl. Acad. Sci. USA.

[28] Whisstock J.C., Lesk A.M. (2003). Prediction of protein function from protein sequence and structure. Q. Rev. Biophys..

[29] Guzzo A.V. (1965). The influence of amino-acid sequence on protein structure. Biophys. J..

[30] Sun T., Zhou B., Lai L., Pei J. (2017). Sequence-based prediction of protein protein interaction using a deep-learning algorithm. BMC Bioinform..

[31] Jones S., Thornton J.M. (1996). Principles of protein-protein interactions. Proc. Natl. Acad. Sci. USA.

[32] Nooren I.M., Thornton J.M. (2003). Diversity of protein-protein interactions. EMBO J..

[33] Obenauer J.C., Yaffe M.B. (2004). Computational prediction of protein-protein interactions. Methods Mol. Biol..

[34] Smith G.R., Sternberg M.J. (2002). Prediction of protein-protein interactions by docking methods. Curr. Opin. Struct. Biol..

[35] Gschwend D.A., Good A.C., Kuntz I.D. (1996). Molecular docking towards drug discovery. J. Mol. Recognit..

[36] Durrant J.D., McCammon J.A. (2011). Molecular dynamics simulations and drug discovery. BMC Biol..

[37] Hiratsuka T. (1994). Nucleotide-induced closure of the ATP-binding pocket in myosin subfragment-1. J. Biol. Chem..

[38] Goodson H.V., Spudich J.A. (1993). Molecular evolution of the myosin family: Relationships derived from comparisons of amino acid sequences. Proc. Natl. Acad. Sci. USA.

[39] Espreafico E.M., Cheney R.E., Matteoli M., Nascimento A.A., De Camilli P.V., Larson R.E., Mooseker M.S. (1992). Primary structure and cellular localization of chicken brain myosin-V (p190), an unconventional myosin with calmodulin light chains. J. Cell Biol..

[40] Mueller H., Perry S.V. (1962). The degradation of heavy meromyosin by trypsin. Biochem. J..

[41] Notredame C., Higgins D.G., Heringa J. (2000). T-Coffee: A novel method for fast and accurate multiple sequence alignment. J. Mol. Biol..

[42] Waterhouse A.M., Procter J.B., Martin D.M., Clamp M., Barton G.J. (2009). Jalview Version 2--a multiple sequence alignment editor and analysis workbench. Bioinformatics.

[43] Fujii T., Namba K. (2017). Structure of actomyosin rigour complex at 5.2 Å resolution and insights into the ATPase cycle mechanism. Nat. Commun..

[44] Waterhouse A., Bertoni M., Bienert S., Studer G., Tauriello G., Gumienny R., Heer F.T., de Beer T.A.P., Rempfer C., Bordoli L. (2018). SWISS-MODEL: Homology modelling of protein structures and complexes. Nucleic Acids Res..

[45] Jumper J., Evans R., Pritzel A., Green T., Figurnov M., Ronneberger O., Tunyasuvunakool K., Bates R., Žídek A., Potapenko A. (2021). Highly accurate protein structure prediction with AlphaFold. Nature.

[46] Behrmann E., Müller M., Penczek P.A., Mannherz H.G., Manstein D.J., Raunser S. (2012). Structure of the rigor actin-tropomyosin-myosin complex. Cell.

[47] Lorenz M., Holmes K.C. (2010). The actin-myosin interface. Proc. Natl. Acad. Sci. USA.

[48] Pospich S., Sweeney H.L., Houdusse A., Raunser S. (2021). High-resolution structures of the actomyosin-V complex in three nucleotide states provide insights into the force generation mechanism. Elife.

[49] Risi C., Schäfer L.U., Belknap B., Pepper I., White H.D., Schröder G.F., Galkin V.E. (2021). High-Resolution Cryo-EM Structure of the Cardiac Actomyosin Complex. Structure.

[50] von der Ecken J., Heissler S.M., Pathan-Chhatbar S., Manstein D.J., Raunser S. (2016). Cryo-EM structure of a human cytoplasmic actomyosin complex at near-atomic resolution. Nature.

[51] Gurel P.S., Kim L.Y., Ruijgrok P.V., Omabegho T., Bryant Z., Alushin G.M. (2017). Cryo-EM structures reveal specialization at the myosin VI-actin interface and a mechanism of force sensitivity. Elife.

[52] Furch M., Remmel B., Geeves M.A., Manstein D.J. (2000). Stabilization of the actomyosin complex by negative charges on myosin. Biochemistry.

[53] Joel P.B., Trybus K.M., Sweeney H.L. (2001). Two conserved lysines at the 50/20-kDa junction of myosin are necessary for triggering actin activation. J. Biol. Chem..

[54] Onishi H., Mikhailenko S.V., Morales M.F. (2006). Toward understanding actin activation of myosin ATPase: The role of myosin surface loops. Proc. Natl. Acad. Sci. USA.

[55] Yan Y., Zhang D., Zhou P., Li B., Huang S.Y. (2017). HDOCK: A web server for protein-protein and protein-DNA/RNA docking based on a hybrid strategy. Nucleic Acids Res..

[56] Risal D., Gourinath S., Himmel D.M., Szent-Györgyi A.G., Cohen C. (2004). Myosin subfragment 1 structures reveal a partially bound nucleotide and a complex salt bridge that helps couple nucleotide and actin binding. Proc. Natl. Acad. Sci. USA.

[57] Coureux P.D., Wells A.L., Ménétrey J., Yengo C.M., Morris C.A., Sweeney H.L., Houdusse A. (2003). A structural state of the myosin V motor without bound nucleotide. Nature.

[58] Ropars V., Yang Z., Isabet T., Blanc F., Zhou K., Lin T., Liu X., Hissier P., Samazan F., Amigues B. (2016). The myosin X motor is optimized for movement on actin bundles. Nat. Commun..

[59] Gibson F., Walsh J., Mburu P., Varela A., Brown K.A., Antonio M., Beisel K.W., Steel K.P., Brown S.D. (1995). A type VII myosin encoded by the mouse deafness gene shaker-1. Nature.

[60] Self T., Mahony M., Fleming J., Walsh J., Brown S.D., Steel K.P. (1998). Shaker-1 mutations reveal roles for myosin VIIA in both development and function of cochlear hair cells. Development.

[61] Liu X., Udovichenko I.P., Brown S.D., Steel K.P., Williams D.S. (1999). Myosin VIIa participates in opsin transport through the photoreceptor cilium. J. Neurosci..

[62] Reubold T.F., Eschenburg S., Becker A., Kull F.J., Manstein D.J. (2003). A structural model for actin-induced nucleotide release in myosin. Nat. Struct. Biol..

[63] Onishi H., Morales M.F., Kojima S., Katoh K., Fujiwara K. (1997). Functional transitions in myosin: Role of highly conserved Gly and Glu residues in the active site. Biochemistry.

[64] Guzik-Lendrum S., Heissler S.M., Billington N., Takagi Y., Yang Y., Knight P.J., Homsher E., Sellers J.R. (2013). Mammalian myosin-18A, a highly divergent myosin. J. Biol. Chem..

[65] Llinas P., Isabet T., Song L., Ropars V., Zong B., Benisty H., Sirigu S., Morris C., Kikuti C., Safer D. (2015). How actin initiates the motor activity of Myosin. Dev. Cell.

[66] Sasaki N., Ohkura R., Sutoh K. (2002). Dictyostelium myosin II as a model to study the actin-myosin interactions during force generation. J. Muscle Res. Cell Motil..

[67] Gong R., Jiang F., Moreland Z.G., Reynolds M.J., de Los Reyes S.E., Gurel P., Shams A., Heidings J.B., Bowl M.R., Bird J.E. (2022). Structural basis for tunable control of actin dynamics by myosin-15 in mechanosensory stereocilia. Sci. Adv..

[68] Morck M.M., Bhowmik D., Pathak D., Dawood A., Spudich J., Ruppel K.M. (2022). Hypertrophic cardiomyopathy mutations in the pliant and light chain-binding regions of the lever arm of human β-cardiac myosin have divergent effects on myosin function. Elife.

[69] Yang Y., Kovács M., Sakamoto T., Zhang F., Kiehart D.P., Sellers J.R. (2006). Dimerized Drosophila myosin VIIa: A processive motor. Proc. Natl. Acad. Sci. USA.

[70] Pettersen E.F., Goddard T.D., Huang C.C., Couch G.S., Greenblatt D.M., Meng E.C., Ferrin T.E. (2004). UCSF Chimera—A visualization system for exploratory research and analysis. J. Comput. Chem..

