# Supplementary material for: Sequence Alignment-Based Prediction of Myosin 7A: Structural Implications and Protein Interactions
Source: Int J Mol Sci. 2024 Mar 16;25(6):3365. doi: 10.3390/ijms25063365 (PMC10970253; doi:10.3390/ijms25063365)
Supplement: Supplementary file 1 [file ijms-25-03365-s001.zip › ijms-2884730-supplementary.pdf]

Supplementary Material

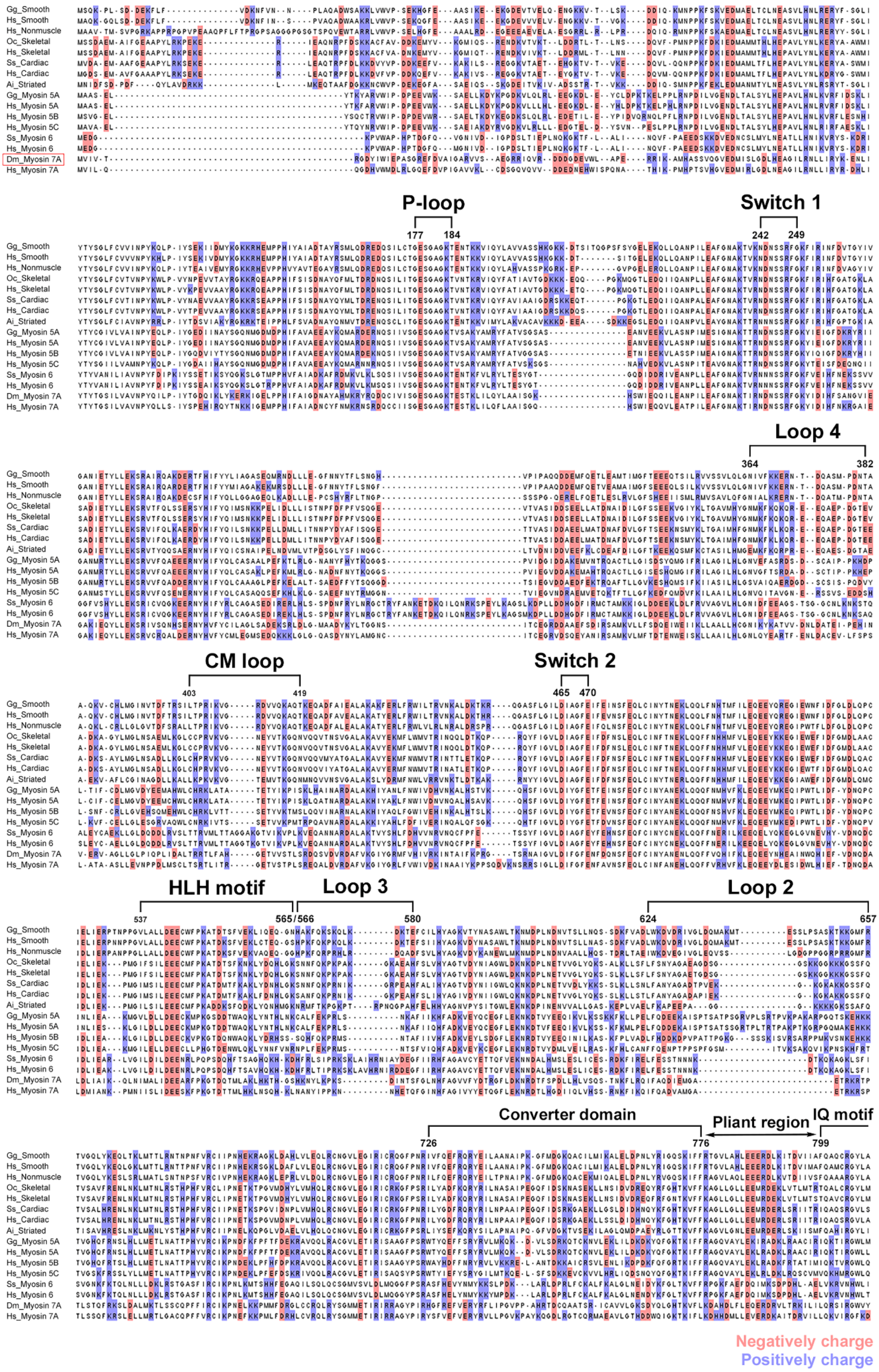

Figure S1 Amino acid sequence alignment of heavy chains from myosin 7A and other myosins. Nucleotide binding sites of myosin 7A: Phosphate loop (G156 – T163), Switch-1 (N205 – G212), and Switch-2 (D431 – E436). Actin binding sites of myosin 7A: Loop 4 (N328 – N349), Cardiomyopathy loop (T369 – S385), Helix-loop-helix motif (I500 – S528), Loop 3 (H529 – S542), Loop 2 (I586 - P602). Converter domain of myosin 7A (H670 -L720). The pliant region is indicated at the junction between the converter domain and lever arm. The residue number in this figure corresponds to the original residues from skeletal myosin 2. Positively and negatively charged residues are shown in red and blue, respectively. Species codes: *Gg Gallus gallus*; *Hs Homo sapiens*; *Oc Oryctolagus cuniculus*; *Ss Sus scrofa*; *Ai Argopecten irradians*; *Dm Drosophila melanogaster*.

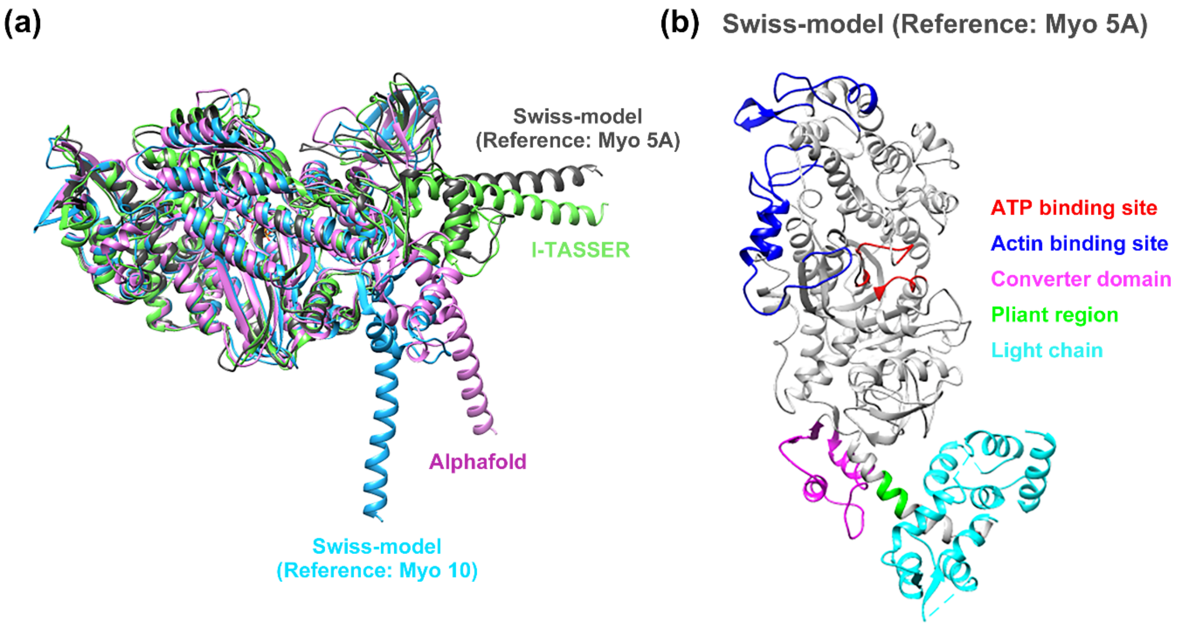

Figure S2 Homology modeling and comparative analysis of myosin 7A using AlphaFold (AF-Q9V3Z6-F1), I-TASSER, and SWISS-MODEL. (a) The modeling results, leveraging differences in software and reference selection, indicate a high structural similarity in the motor domain, with the exception of the flexible regions around the neck area. (b) Utilized a homology model based on the reference of the representative unconventional myosin, myosin 5A. The key active sites shown as different colors respectively.

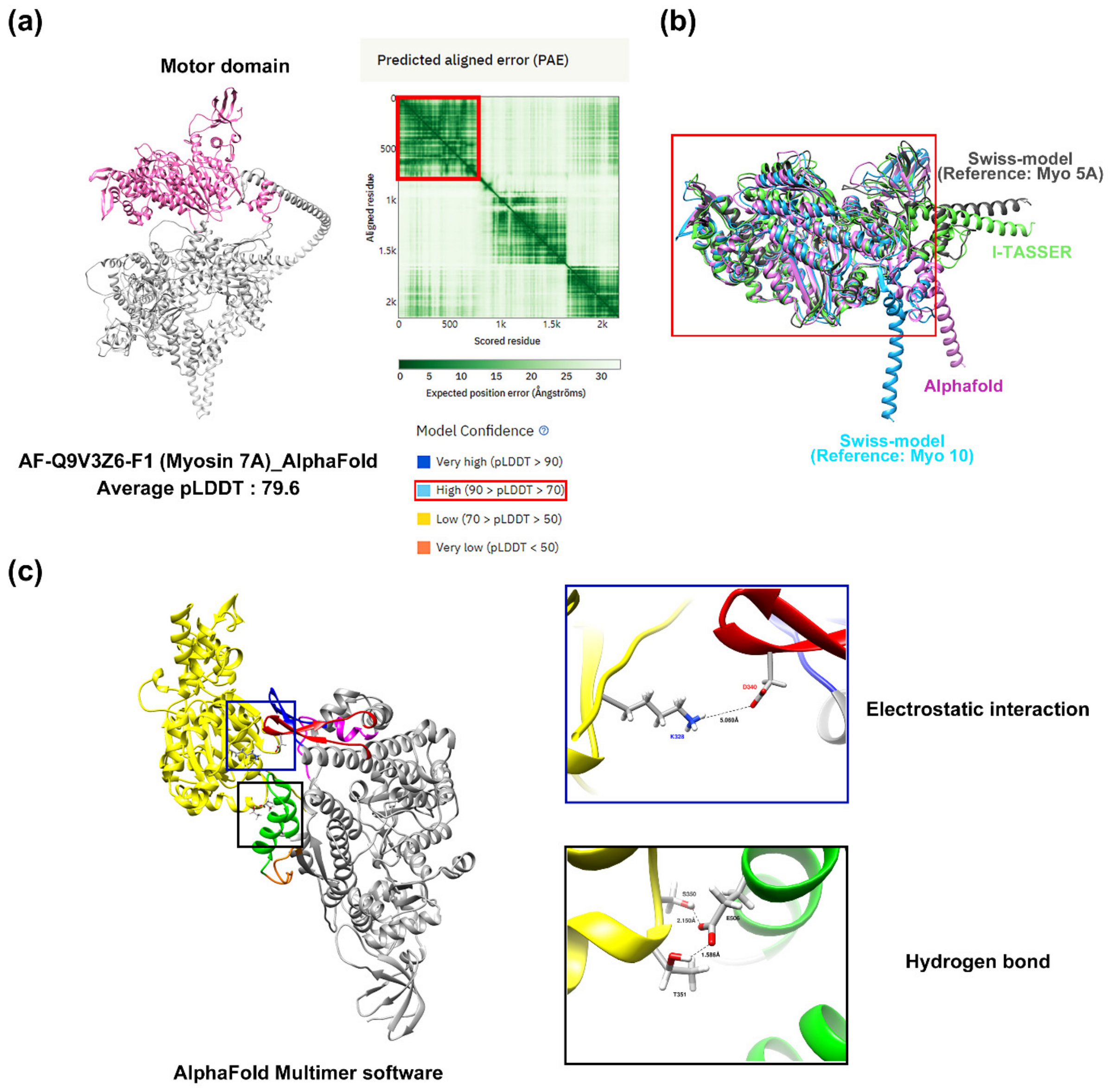

Figure S3 Assessment of Prediction Models: Evaluating Structural Integrity. (a) The Myosin 7A AlphaFold structure (Uniprot: Q9V3Z6) has very low predicted aligned error (PAE) and per-residue local distance difference test (pLDDT) values in its motor domain, indicating high confidence in the model's reliability. (b) Comparing myosin 7A homology models generated by other software (SWISS-MODEL and I-TASSER) with the AlphaFold model confirms their high structural similarity in the motor domain, ensuring the reliability of all predicted homology models. (c) The AlphaFold Multimer software predicted the actomyosin-7A structure, revealing the same core interaction between actin and myosin 7A as previously proposed by HDOCK software. The structural predictions for *Drosophila* myosin 7A and alpha skeletal actin were acquired from the AlphaFold protein structure database. These predictions were generated by submitting the respective protein sequences to AlphaFold, utilizing default settings without template assistance. Subsequently, the final structural complex was visualized using UCSF Chimera for comprehensive analysis.
